# Changes of serum uric acid level during acute gout flare and related factors

**DOI:** 10.3389/fendo.2023.1077059

**Published:** 2023-02-21

**Authors:** Jie Zhang, Wenyan Sun, Fei Gao, Jie Lu, Kelei Li, Yijun Xu, Yushuang Li, Changgui Li, Ying Chen

**Affiliations:** ^1^ Department of Endocrinology and Metabolism, The Affiliated Hospital of Qingdao University, Qingdao, China; ^2^ Department of Endocrinology and Metabolism, Xuanwu Hospital, Capital Medical University, Beijing, China; ^3^ Department of Hand and Foot Surgery, The Affiliated Hospital of Qingdao University, Qingdao, China; ^4^ Shandong Provincial Key Laboratory of Metabolic Diseases and Qingdao Key Laboratory of Gout, The Affiliated Hospital of Qingdao University, Qingdao, China; ^5^ Institute of Metabolic Diseases, Qingdao University, Qingdao, China; ^6^ Institute of Nutrition and Health, Qingdao University, Qingdao, China; ^7^ Shandong Provincial Clinical Research Center for Immune Diseases and Gout, Qingdao, China

**Keywords:** acute gouty arthritis, uric acid, urinary uric acid excretion, cortisol, inflammation

## Abstract

**Objective:**

By studying the changes of serum uric acid (SUA) in acute stage and remission stage of gouty arthritis, we aimed to explore the relationship between the changes of SUA level and free glucocorticoids and inflammatory factors.

**Methods:**

A prospective, longitudinal study was conducted on 50 acute gout patients in the dedicated gout clinic of the Affiliated Hospital of Qingdao University. Blood and 24-hour urine samples were collected during the acute phase and two weeks after the initial visit. Patients with acute gouty arthritis were treated primarily with colchicine and nonsteroidal anti-inflammatory drugs.

**Results:**

A total of 32 patients completed the two-week follow-up trial. SUA levels were significantly downregulated during the acute flare than after the flare (464.14 ± 90.97 *vs*. 527.36 ± 86.90 μmol/L, *p* < 0.001). The 24-hour fractional excretion of uric acid (24 h FEur) (5.54 ± 2.82% *vs*. 4.68 ± 2.83%, *p* < 0.001) and 24-hour urinary uric acid excretion (24 h Uur) (663.08 ± 249.48 μmol/L *vs*. 540.87 ± 263.18 μmol/L, *p* = 0.001) increased significantly in patients during the acute phase. The percent change in SUA was associated with those in 24 h FEur and C-reactive protein. Meanwhile, the percent change in 24 h Uur was associated with those in 24-hour urinary free cortisol, percent change in interleukin 1β and interleukin 6.

**Conclusion:**

Decreased SUA level during the acute gout flare was associated with increased excretion of urinary uric acid. Inflammatory factors and bioactive free glucocorticoids may play significant roles in this process.

## Introduction

Gout is caused by prolonged hyperuricemia, which can result in monosodium urate formation and deposition in the joint and other tissues, potentially leading to acute gouty arthritis (1). Hyperuricemia is not only a pathogenic factor for gout onset but also an important indicator of gout diagnosis. Serum uric acid (SUA) level is a significant predictor of gout flare and related costs (2). However, gouty arthritis may also develop in individuals with normal SUA levels under some specific conditions.

The SUA level is typically within the normal range during the acute gout phase (3). The recently updated European League Against Rheumatism guideline emphasizes that the SUA level is insufficient to diagnose gout during an acute flare and should preferably be determined at a distance from the flare due to its limited diagnostic value (4). According to general consensus, the diagnosis of gout should not be based on the SUA level alone but be systematically assessed considering the symptoms and imaging evidence, including synovial fluid analysis.

Normally, urate elimination occurs *via* two routes: renal elimination and extrarenal elimination, and approximately 65% of the daily urate production is excreted *via* the urinary route (5). A recent study indicated that SUA decreases during acute gouty arthritis, which might be associated with increased urinary excretion of uric acid (6). Previous research has also indicated that the inflammatory process is crucial in the mechanism of increased uric acid excretion during acute gouty arthritis (7).

Nevertheless, the changes of the SUA level fluctuated greatly, and the research methods were different, there is a lack of high-quality prospective research. Besides, there is also disagreement about the effect of inflammation on uric acid levels. Thus, this study aimed to evaluate the change in uric acid level during the acute phase and explore the relationship between uric acid level, glucocorticoids, and inflammatory factors, providing additional evidence for clinical practice.

## Materials and methods

### Patients

Patients were recruited from the outpatients who visited the dedicated Gout Clinic of the Affiliated Hospital of Qingdao University, and the trial was registered on the Chinese Clinical Trials Registry as #ChiCTR2000038794. The Medical Ethics Committee of the Affiliated Hospital of Qingdao University approved this study. Before the study began, all participants received an adequate explanation of the study’s objectives and provided written informed consent.

All patients were diagnosed with acute gouty arthritis based on clinical criteria, including laboratory and imaging examinations. They had not received any urate-lowering drugs within two weeks before the interview for enrollment in this study. Patients who were taking corticosteroids, anticoagulants, diuretics, or other drugs that alter uric acid excretion were excluded from the study. Other exclusion criteria included patients with hepatic insufficiency (alanine aminotransferase [ALT] or aspartate aminotransferase [AST] > 1.5 times the upper limit of normal; serum total bilirubin [TBIL] > 2 times the upper limit of normal), renal insufficiency (patients with severe renal injury or end-stage renal disease; estimated glomerular filtration rate [eGFR] < 30 mL/min/1.73 m^2^), active peptic ulcer combined with heart disease, malignant tumors, active tuberculosis or blood disease, and the judgment of the investigator that the candidate was inappropriate for this research.

### Study design

This was a prospective, longitudinal study. Patients enrolled in the study were treated primarily with colchicine and nonsteroidal anti-inflammatory drugs (NSAIDs) for one week, with a corticosteroid administered if severe pain was present. The patients were instructed to avoid alcohol or high-purine foods until their next visit to the hospital. We performed clinical and biochemical assessments on patients with acute gouty arthritis and after the flare. Blood and 24-hour urine samples were collected at two different timepoints, i.e., during the flare and two weeks after the initial visit.

At baseline, the following data were obtained: age, sex, disease history, height, body weight, blood pressure, family history, and tophi. Body mass index was calculated by dividing weight (kg) by height in meters squared (m^2^). At the aforementioned timepoints, we collected blood samples to assay the SUA, serum cortisol (CORT), C-reactive protein (CRP), interleukin (IL)-1β, IL-6, and tumor necrosis factor-alpha (TNF-α), whereas 24-hour urine samples were collected to assay 24-hour urinary uric acid excretion (24 h Uur), 24-hour fractional excretion of uric acid (24 h FEur), excretion of uric acid per volume of glomerular filtration (EurGF), and 24-hour urinary free cortisol (24 h UFC) concentration. We measured the pH value using sanguinis urine samples. The 24 h FEur represents uric acid clearance per creatinine clearance and can be calculated as 24 h FEur = (24 h urinary uric acid/SUA) × (serum creatinine/24 h urinary creatinine) × 100, expressed as a percentage. We calculated the EurGF as follows: EurGF = (urinary uric × serum creatinine)/urinary creatinine, expressed in mg/dL of glomerular filtration; GFR=[(140-age) × weight]/(0.818 × serum creatinine). We normalized 24 h Uur for a body surface area of 1.73 m^2^.

The concentrations of the cytokines IL-1β, IL-6, and TNF-α in serum were estimated using a validated enzyme-linked immunosorbent assay and a Human Inflammatory Cytokines Kit (Elabscience Biotechnology Co., Ltd, China). At the two timepoints, we measured other biochemical parameters, such as serum ALT, serum AST, serum glucose (GLU), serum triglyceride (TG), total cholesterol (TC), urea nitrogen (BUN), and serum creatinine (SCr) using an automatic biochemical analyzer (TBA-40FR, Toshiba, Japan).

### Statistical analysis

Data with normal distribution are expressed as mean ± SD, whereas data with abnormal distribution are expressed as median (interquartile range [IQR]). We employed a paired t-test to compare changes in parameters during and after the gout flare. The differences between the groups were tested for statistical significance by an independent sample t-test. The Mann–Whitney *U* non-parametric test was employed for variables that were not normally distributed. We calculated Pearson’s correlation coefficient to evaluate the relationship between parameters. We tested the association between SUA and the levels of CORT, IL-1β, IL-6, and TNF-α using the same model. Further, we applied linear mixed effect models to estimate the association between the percent changes in 24 h Uur and 24 h UFC. Model 1 required no adjustment. Model 2 was adjusted for the percent changes in IL-1β, IL-6, and TNF-α. Model 3 was further adjusted for the percent changes in CORT, 24 h FEur, and 24 h EurGF. The statistical significance was set at *p* < 0.05, as indicated (two-tailed test). SPSS v.26.0 (IBM SPSS, Chicago, IL, USA) was used for data analysis.

## Results

### Patients’ enrollment and characteristics

**Figure 1:**
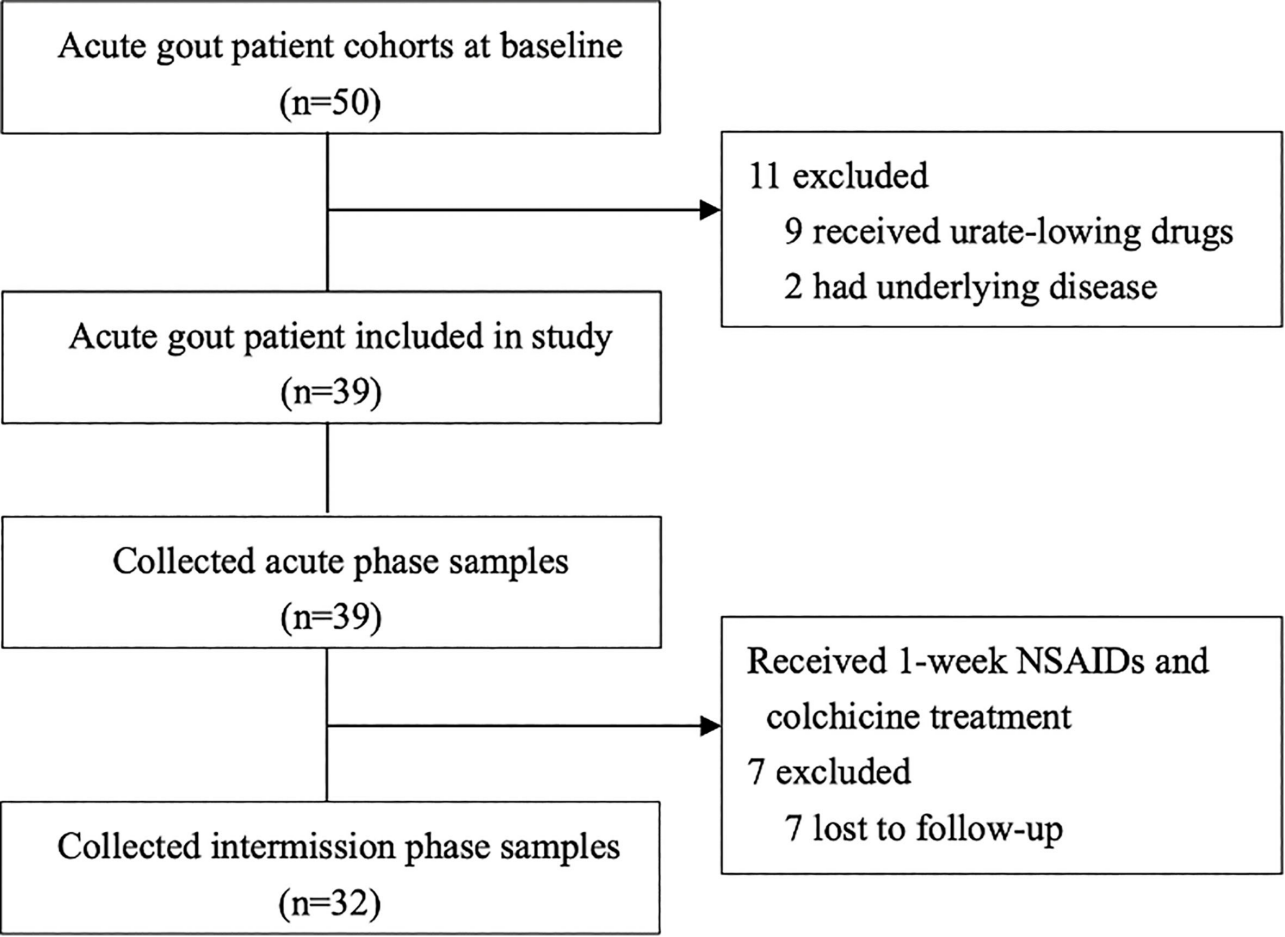
Flow chart of the study.

From October 2020 to September 2021, 32 participants (age 19–73 years, mean ± SD 41.68 ± 14.62 years) were enrolled in this study. **Figure 1** depicts the research procedure. Among them, 6 patients had tophi, 5 patients had a family history of gout or hyperuricemia, 14 patients had hypertension, 1 patient had diabetes mellitus, 13 patients had fatty liver, 12 patients had hyperlipidemia, and 2 patients had kidney stones.

### Serum urate and uric acid excretion

**Figure 2:**
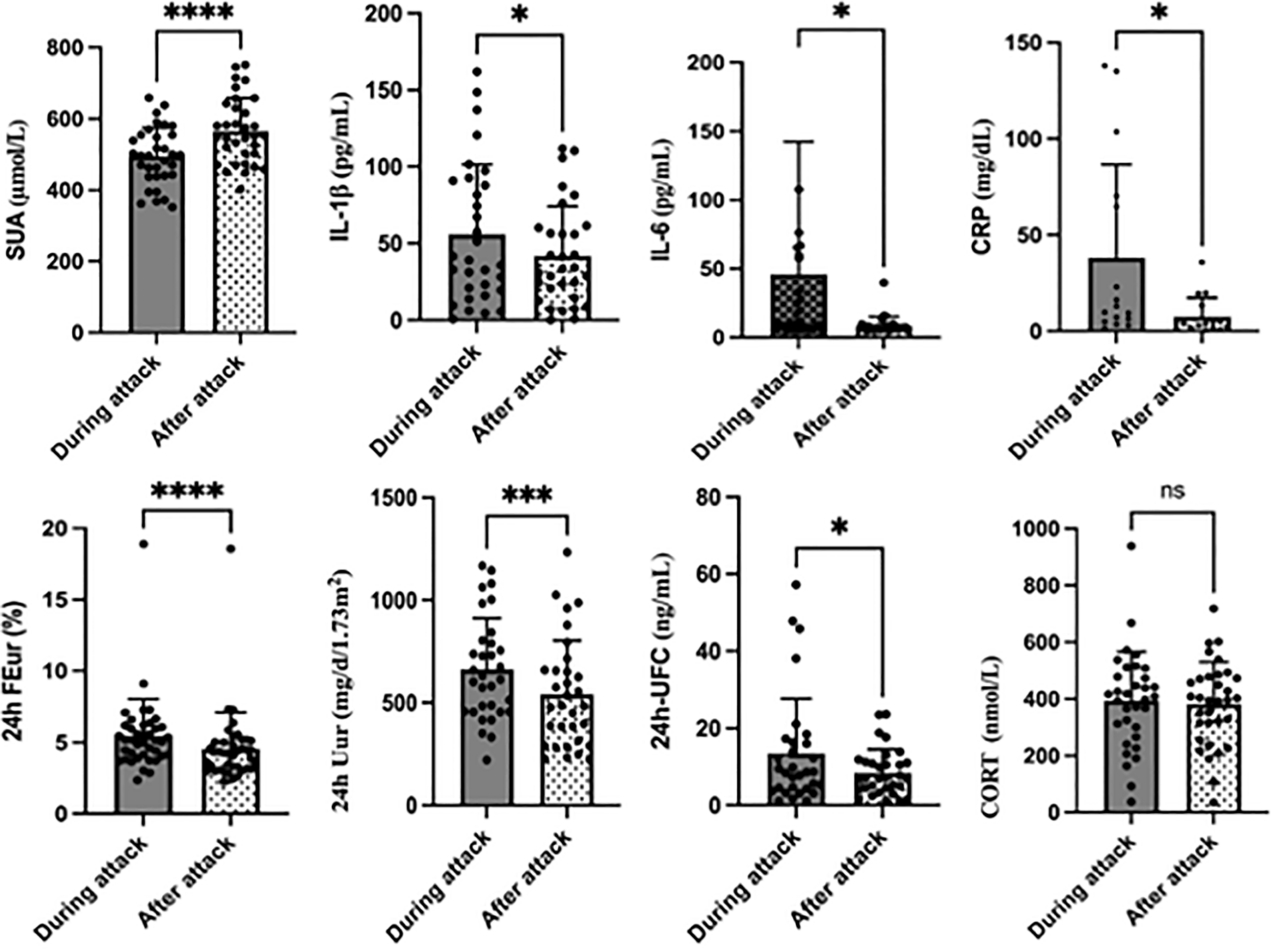
Changes in primary outcomes during and after acute gouty attack. *P< 0.05, **P<0.01, ***P<0.001,****P<0.0001, ns, no significance.

As shown in **Table 1**, the SUA level in patients during acute gouty flare significantly decreased by 12% from a mean value of 527.36 μmol/L to 464.14 μmol/L compared with that in the interval period (*p* < 0.001). This was accompanied by an increase in both fractional excretion of 24 h FEur (5.54 ± 2.82% *vs*. 4.68 ± 2.83%, *p* < 0.001) and 24 h Uur excretion (663.08 ± 249.48 mg/d/1.73 m^2^ *vs*. 540.87 ± 263.18 mg/d/1.73 m^2^, *p* = 0.001) in the acute period compared with those in the interval period (**Figure 2**). Furthermore, the 24 h EurGF was increased (28.18 ± 17.13 *vs*. 25.00 ± 12.86, *p* = 0.012) during the acute phase. In this study, hyperuricemia was diagnosed when the SUA level was more than 420 μmol/L. Thus, during the acute gout flare, 10 of the 32 patients (31.25%) had a normal SUA level.

**Table 1:** Biochemical parameters during and after acute gouty attack.

	During attack (N=32)	After attack (N=32)	*P*-value
Serum			
ALT (U/L)	34.80 ± 18.57	37.02 ± 24.43	0.076
AST (U/L)	23.39 ± 11.77	23.50 ± 8.22	0.891
GLU (mmol/L)	6.00 ± 1.49	5.73 ± 0.96	0.002
TG (mmol/L)	2.19 ± 1.56	2.25 ± 1.40	0.588
TC (mmol/L)	4.91 ± 1.09	4.91 ± 1.01	0.960
BUN (mmol/L)	4.66 ± 1.20	4.73 ± 1.14	0.355
Scr (μmol/L)	83.34 ± 18.86	85.23 ± 13.85	0.003
SUA (μmol/L)	464.14 ± 90.97	527.36 ± 86.90	<0.001
GFR (mL/min)	126.15 ± 39.43	122.83 ± 36.72	<0.001
CRP (mg/dL)	38.12 ± 48.46	7.55 ± 9.82	0.014
CORT (nmol/L)	394.15 ± 172.49	382.22 ± 147.69	0.676
IL-1β (pg/mL)	55.92 ± 45.54	41.86 ± 32.42	0.028
IL-6 (pg/mL)	45.89 ± 96.51	8.75 ± 6.43	0.031
TNF-α (pg/mL)	16.14 ± 6.33	18.24 ± 8.53	0.075
Urine			
24h Uur (mg/d/1.73m^2^)	663.08 ± 249.48	540.87 ± 263.18	0.001
24h EurGF (mg/d/1.73m^2^)	28.18 ± 17.13	25.00 ± 12.86	0.012
24h FEur (%)	5.54 ± 2.82	4.68 ± 2.83	< 0.001
24h UFC (ng/mL)	13.41 ± 14.22	8.46 ± 6.07	0.039
pH	5.77 ± 0.58	5.67 ± 0.50	0.099

Data are mean ± SD. ALT: alanine aminotransferase, AST: aspartate aminotransferase, GLU: fasting plasma glucose, TG: triglycerides, TC: cholesterol, BUN: urea nitrogen, Scr: serum creatinine, SUA: serum uric acid, CRP: C-reactive protein, CORT: serum cortisol, IL-1β: interleukin 1β, IL-6: interleukin 6, TNF-α: tumor necrosis factor α, 24h Uur: 24-hour urinary uric acid excretion, 24h EurGF: excretion of uric acid per volume of glomerular filtration, 24h FEur: 24-hour fractional excretion of uric acid, 24h UFC: 24-hour urinary free cortisol.

To explore the differences between the normal SUA group and the hyperuricemia group, we divided the patients into two groups based on the SUA level during the acute phase: SUA < 420 μmol/L and SUA ≥ 420 μmol/L groups. In the group with SUA < 420 μmol/L, CRP, changes in CRP and 24 h FEur in patients were higher, whereas SCr was lower than in the hyperuricemia group (**Table 2**). Furthermore, the patients with higher SUA levels were younger.

**Table 2:** Comparison in different SUA level during the acute gout phase.

	SUA <420μmol/L (N=10)	SUA ≥ 420 μmol/L (N=22)	*P*-value
Demographic indicator			
Age (years)	48.21 ± 14.34	39.28 ± 14.06	< 0.001
BMI (kg/m²)	27.21 ± 3.07	27.55 ± 3.65	0.523
Tophus	9.5%	7.6%	0.654
Diabetes	15.9%	11.5%	0.389
Kidney stone	3.2%	8.4%	0.291
Family history	14.3%	19.1%	0.410
Inflammation indicators			
CRP ^*^(mg/dL)	26.76 ± 39.47	6.88 ± 3.80	0.012
CORT ^*^ (nmol/L)	354.83 ± 109.28	403.39 ± 185.90	0.406
IL-1β ^*^ (pg/mL)	73.54 ± 44.11	46.23 ± 44.41	0.111
IL-6 ^*^ (pg/mL)	67.57 ± 146.94	33.97 ± 54.04	0.363
TNF-α ^*^ (pg/mL)	17.96 ± 7.91	15.14 ± 5.23	0.241
ΔCRP (mg/dL)	41.99 ± 49.06	6.22 ± 2.72	0.036
Urine excretion			
Scr ^*^ (μmol/L)	79.35 ± 13.92	86.53 ± 17.37	0.005
24h Uur ^*^ (mg/d/1.73m^2^)	586.06 ± 185.80	5673.09 ± 250.67	0.365
24h EurGF ^*^ (mg/d/1.73m^2^)	23.50 ± 1.60	29.05 ± 18,56	0.515
24h FEur ^*^ (%)	6.19 ± 1.17	5.28 ± 2.81	0.381
24h UFC ^*^(ng/mL)	9.52 ± 5.55	14.13 ± 15.26	0.514
Δ24h FEur(%)	2.25 ± 1.21	0.60 ± 0.90	0.001
pH	5.73 ± 0.59	5.79 ± 0.59	0.531

Data are mean ± SD (number). *: indicator in the acute phase, BMI: body mass index, Scr: serum creatinine, CRP: C-reactive protein, CORT: serum cortisol, IL-1β: interleukin 1β, IL-6: interleukin 6, TNF-α: tumor necrosis factor α, ΔCRP: change of C-reactive protein, 24h Uur: 24-hour urinary uric acid excretion, 24h EurGF: excretion of uric acid per volume of glomerular filtration, 24h FEur: 24-hour fractional excretion of uric acid, 24h UFC: 24-hour urinary free cortisol.

### Cortisol and inflammatory indicators

As shown in **Table 1** and **Figure 2**, the 24 h UFC concentration increased significantly in the acute gout phase than in the interval phase (13.41 ± 14.22 *vs*. 8.46 ± 6.07, *p* = 0.039). There was also a slight increase in CORT, but without a statistical difference (*p* = 0.676). Regarding the inflammatory indicators, there was a significant increase in the levels of CRP (38.12 ± 48.46 *vs*. 7.55 ± 9.82, *p* = 0.014), IL-1β (55.92 ± 45.54 *vs*. 41.86 ± 32.42, *p* = 0.028) and IL-6 (45.89 ± 96.51 *vs*. 8.75 ± 6.43, *p* = 0.031) when the patient had an acute gout flare.

**Table 3** shows the correlation matrix among the percent changes in SUA, 24 h Uur, FEur, CRP, IL-1β, IL-6, CORT, and 24 h UFC to determine the relationship between these variables according to Pearson’s correlation. The percent change in SUA was associated with those in CRP (r = 0.245, *p* = 0.016) and 24 h FEur (r = −0.644, *p* < 0.001). The percent change in CRP was associated with those in 24 h FEur (r = 0.019, *p* = 0.022), 24 h Uur (r = 0.651, *p* = 0.016), CORT (r = 0.840, *p* = 0.001), and 24 h UFC (r = 0.869, *p* < 0.001). The percent change in 24 h Uur was associated with those in CRP (r = 0.651, *p* =0.016, IL-1β (r = 0.507, *p* = 0.012), IL-6 (r = 0.745, *p* < 0.001), CORT (r = 0.452, *p* = 0.035) and 24 h UFC (r = 0.438, *p* = 0.012). Meanwhile, the percent change in 24 h UFC was associated with those in CRP (r = 0.869, *p* < 0.001), IL-1β (r = 0.622, *p* = 0.003, IL-6 (r = 0.940, *p* < 0.001), and CORT (r = 0.600, *p* = 0.003).

**Table 3:** The correlation between uric acid excretion and inflammation.

	Percent change in 24h FEur	Percent change in 24h Uur	SUA during attack	SUA after attack	Percent change in SUA	Percent change in 24h UFC	Percent change in CORT
Percent change in CRP	-0.019^*^	0.651^*^	-0.007	-0.177	0.245^*^	0.869^***^	0.840^**^
Percent change in IL-1β	-0.081	0.507^*^	-0.038	-0.029	0.014	0.622^**^	0.080
Percent change in IL-6	-0.160	0.745^***^	-0.068	-0.317	0.234	0.940^***^	0.527^**^
Percent change in CORT	0.008	0.452^*^	0.089	0.042	0.110	0.600^**^	
Percent change in 24h UFC	-0.120	0.438^*^	-0.031	-0.145	0.306		
Percent change in SUA	-0.644^***^	-0.047					
SUA during attack	-0.370^*^	-0.231					
SUA after attack	0.190	-0.175					

*P< 0.05, **P<0.01, ***P<0.001.

We conducted linear mixed effect models and discovered a significant relationship between the percent changes in 24 h Uur and 24 h UFC. The relationship remained significant after adjusting for the percent changes in IL-1β, IL-6, TNF-α, CORT, 24 h FEur, and 24 h EurGF (β = 0.027, *p* = 0.038) (**Table 4**).

**Table 4:** Linear regression analysis between percent change in 24h Uur and change in 24h UFC in patients.

Models	Percent change in 24h Uur with percent change in 24h UFC	
	Coefficient	P value
Model1	0.076	0.000
Model2	0.025	0.007
Model3	0.027	0.038

Model1, no adjustment; Model2, adjustment for percent change in IL-1β, IL-6, TNF-α; Model 3, additionally adjustment for percent change in CORT and percent change in 24h FEur, 24h EurGF.

### Other biochemical parameters

There were no significant changes in ALT, AST, TG, TC, and BUN levels in serum samples or pH of sanguinis urine samples between the acute and interval periods. However, there was a significant increase in GLU (6.00 ± 1.49 *vs*. 5.73 ± 0.96, *p* = 0.002) in the acute gout phase. Besides, an significant increase in SCr (83.34 ± 18.86 *vs*. 85.23 ± 13.85, *p* = 0.003) and a remarkable decrease in GFR (126.15 ± 39.43 *vs*. 122.83 ± 36.72) after two weeks compared by the initial visit.

## Discussion

In this study, our results indicated that the SUA level was downregulated during an acute gout arthritis flare and was accompanied by a simultaneous increase in uric acid excretion (24 h FEur and 24 h Uur), cortisol levels (24 h UFC concentration), and inflammatory indicators (CRP, IL-1β, and IL-6). In addition, the percent change in SUA was associated with those in 24 h FEur and CRP, whereas the percent change in 24 h Uur was associated with those in 24 h UFC, IL-1β, and IL-6.

The 24 h Uur evaluated the exact uric acid output from the urinary system for 24 hours, while the FEur represented renal function for uric acid excretion. Age, sex, dietary purine intake, circadian rhythm, urinary collection, and technical concerns are factors that may affect the accurate evaluation of normal urinary uric acid excretion (8). By assessing the relationship between SUA, 24 h FEur, 24 h Uur, uric acid excretion/unit GFR, uric acid/creatine ratio, and clearance of urate (Cur), the results indicated that the best relationship in the study was between serum urate and 24 h Uur corrected for body surface area (8). As there were differences in FEur and 24 h Uur excretion during and after the gout flare, it seemed that increased renal uric acid excretion was a potential mechanism for the decreased SUA level during the acute flare.

Gout is a chronic disease. Activation of the NOD-like receptor superfamily pyrin domain-containing 3 inflammasome and release of IL-1β play critical roles in the initiation of acute gout flares (1). Hence, gout has been recognized as a prototype I IL-1β mediated autoinflammatory disease (9). Renal uric acid reabsorption is primarily mediated by two transporters: urate transporter 1 (URAT1) (10) and glucose transporter 9 (GLUT9) (11). ATP-binding cassette transporter, sub-family G, member 2 (ABCG2) has been identified as a high-capacity urate exporter, which could mediate renal and/or extrarenal uric acid excretion (12). A recent study reported that after treatment with IL-1β, an improved excretion of uric acid in human renal proximal tubular epithelial cells (HK-2 cells) was discovered, which was similar to gout patients’ urinary urate excretion during the gout flare (13). In another study, the mice showed increased SUA levels and renal urate excretion but decreased intestinal urate excretion after knocking out ABCG2 (12). Thus, when gout patients experienced an acute flare, it is unclear whether changes in urate excretion also involved URAT1 and GLUT9, and whether changes in those two modes of transport were mediated by IL-1β or other inflammatory factors.

Gout is an autoinflammatory disease that is considered to be associated with IL-6 (14). A previous clinical study discovered that SUA levels decreased when patients received treatment recombined with IL-6, and the study also discovered a concomitant increased renal urate excretion (15). Our results also indicated that IL-6 was associated with urate excretion. We speculated that IL-6 might affect the modulation of urate excretion during the acute phase.

A previous study indicated that acute monoarthritis could induce the activation of the hypothalamic–pituitary–adrenal (HPA) axis, leading to increased expression levels of related genes and concurrent with the increase in plasma CORT (16). Prolonged inflammation would sensitize the pain system and HPA axis, where cortisol likely contributes to pain development (17). IL-6 and TNF-α share HPA-activating activity, although they are less potent and less effective than IL-1 (18).

A previous study confirmed that endogenous bioactive free glucocorticoids appeared to affect urinary excretion rates of uric acid even under physiological conditions (19). A study of nephrolithiasis in Cushing’s disease (CD) discovered increased urinary uric acid excretion in active CD patients due to excessive endogenous glucocorticoid stimulation (20). A study based on a patient with isolated adrenocorticotropin (ACTH) deficiency observed that increased urinary urate excretion might be a direct effect of corticoids on urate transport in the renal tubule (21). A recent study on mice demonstrated that low doses of dexamethasone could increase hepatic uric acid concentration and urinary uric acid excretion, which were associated with the induction of xanthine oxidoreductase in the liver and URAT1 downregulation in the kidney (22).

Furthermore, this study identified a relationship between 24 h Uur, cortisol levels, and inflammatory factors, indicating that cortisol and inflammatory factors influence the process of downregulating SUA levels during the acute phase. We hypothesized that decreased SUA levels during the acute phase were related to the increase of cortisol secretion and urinary uric acid excretion. A previous study also suggested that the inflammatory process contributed to the decreased SUA concentration (6).

Compared with the acute gouty arthritis phase, SCr levels slightly increased two weeks after the initial visit. Treatment with colchicine and NSAIDs, both of which influence renal function, during the acute phase may be the cause behind the increased SCr levels (23, 24). The serum glucose level was higher during the acute phase due to stress hyperglycemia, induced by the collective action of the HPA axis, sympathoadrenal system, and proinflammatory cytokines (25).

We collected 24-hour urinary samples to measure the cortisol level, which allowed us to eliminate the impact of cortisol circadian rhythm on the results. Our study is a prospective cohort study. By comparing the differences of SUA, inflammation index and urinary urate excretion of the same subject at the acute and remission stage, the influence of multiple confounding factors such as age, renal function and comorbidity could be avoided and the results could be more representative. However, it’s inevitable that participants were treated during the acute gout flare phase with NSAIDs which were known to impact GFR. A few patients administered corticosteroid if severe pain was presented might have played the role in increasing uric acid excretion or other potential contributors to observation, which may need further researches to verify. The small size of our sample is also a limitation of our study.

## Conclusion

Decreased SUA level during acute gout flare is associated with increased excretion of urinary uric acid. Inflammatory factors and bioactive free glucocorticoids may play significant roles in this process; whereas, further studies need to be conducted to understand the mechanism.

## Data availability statement

The original contributions presented in the study are included in the article/supplementary material. Further inquiries can be directed to the corresponding authors.

## Ethics statement

The studies involving human participants were reviewed and approved by Medical Ethics Committee of the Affiliated Hospital of Qingdao University. The patients/participants provided their written informed consent to participate in this study.

## Author contributions

JZ was a major contributor in writing the manuscript; JZ, WS, and FG have contributed in the arrangement of follow-up visit; JL and KL have worked on the sample processing; YL and YX have contributed in the interpretation of data; YC were the designers of the study; CGL and YC contributed in revising the manuscript. All authors contributed to the article and approved the submitted version.
